# A Small Effort for Researchers, a Big Gain for Soil Metaproteomics

**DOI:** 10.3389/fmicb.2020.00088

**Published:** 2020-02-04

**Authors:** Marco Chiapello, Elisa Zampieri, Antonietta Mello

**Affiliations:** ^1^Institute for Sustainable Plant Protection, National Research Council, Turin, Italy; ^2^Council for Agricultural Research and Economics Research Centre for Cereal and Industrial Crops (CREA-CI), Vercelli, Italy

**Keywords:** soil metaproteomics, environment, data sharing, PRIDE, microbial communities

The soil is one of the most complex systems where species belonging to different kingdoms live together (Young and Crawford, 2004). In particular, microorganisms such as bacteria and fungi are involved in nutrient cycling and organic matter transformation (Qian and Hettich, 2017): microbial community members with their activities determine nitrogen, sulfur, and carbon fluxes in the terrestrial subsurface (Hug et al., 2016). The soil microbiota with plant growth promoting rhizobacteria (PGPR), P-solubilizing bacteria, mycorrhizal-helping bacteria (MHB), and arbuscular mycorrhizal fungi (AMF) can be moreover involved in transfer and mobilization of trace elements, allowing bioremediation of heavy metal contaminated fields (Khan, 2005) or oil-polluted Alpine soils (Margesin, 2000). Microorganisms can be considered as tools to remove pollutants in soil, water, and sediments (Abatenh et al., 2017).

In order to understand the soil ecosystems and the biological processes that characterize it, it is necessary to study its microbial community composition, but also the metabolic activities performed by microbes (Siggins et al., 2012; Mello and Zampieri, 2017). The metagenomics advent made possible to identify the microorganism communities present in the soil (Vogel et al., 2009), while metaproteomics made possible to investigate the biological functions of these communities (Qian and Hettich, 2017). When the two approaches are applied to the same target it becomes possible to link microbial community composition to ecological processes, as performed by Zampieri et al. (2016) who tried to decipher the functioning of the brulé, the particular niche where a fungus in symbiosis with forest trees drives out the other symbiotic fungi. Recently, Martinez-Alonso et al. (2019) combined different -omic techniques (16S rRNA sequencing, culturomics, and metaproteomics) in order to identify microbial species and to clarify functions of microbial populations in the englacial ecosystem.

The study of the proteins expressed in an ecosystem at a specific time is a hot topic. Considering only soil ecosystem, using “metaproteomics AND soil” as topics on Web of Science website (http://www.webofknowledge.com/), it was possible to find 151 papers (as on September 15th 2019). Among the soil related papers according to Web of Science classification, there are: 36 reviews, 9 book chapters, 4 meetings, 2 abstracts, 1 letter, 1 early access, 71 others, and 148 articles.

Although the metaproteomics technique is more than 10 years old, it is still challenged both by technical and computational limitations. Humic acids and other contaminants which interfere with the protein extraction, render it highly dependent on the soil type. Different extraction methods can influence the observed metaproteome (Taylor and Williams, 2010; Becher et al., 2013; Zampieri et al., 2016; Mattarozzi et al., 2017; Keiblinger and Riedel, 2018). A strategy to overcome this obstacle is to use parallel different extraction methods and pool the extracted proteins before the subsequent analysis. The absence of complete protein databases (Wilmes and Bond, 2006; Bastida et al., 2009; Siggins et al., 2012; Becher et al., 2013; Wilmes et al., 2015; Keiblinger et al., 2016; Wang et al., 2016; Heyer et al., 2017; Callister et al., 2018; Starke et al., 2019) limits the protein identification. A promising solution is to build in-house databases based on metagenomics data previously obtained from the same environment (Zampieri et al., 2016; Mattarozzi et al., 2017). The combination of metagenomics and metaproteomics has surely received some help by the recent advent of inexpensive high-throughput sequencing (Wilmes et al., 2015); by next generation sequencing it is in fact possible to obtain in less time more reads that can allow the organism identification and can create a starting point for building a database tailored for protein identification.

Despite all the limitations, the metaproteomics is a powerful technique to study the biological functions of microbial communities, to correlate the taxonomic and functional soil composition within the environment (Heyer et al., 2017) and also to evaluate the responses of microbial communities to climate change (i.e., global warming) (Liu et al., 2017). Moreover, soil protein identification could give information about the soil biogeochemical potential and pollutant degradation and be an indicator of soil quality (Bastida et al., 2019) and regeneration.

Looking at the 148 articles found in Web of Science website, a consistent number of studies has been done all around the world, on different soil types, such as agricultural, forest, contaminated by heavy metals, desert, riparian ones. The metaproteomic analyses were principally carried out in the Northern hemisphere (with the exception of few works in the Southern part of Africa). While in the USA the studied matrix is associated mainly with water, in Asia, basically in China, it is mostly associated with crops. Considering Europe, the studied soil types are heterogeneous, characterized by prevalence of forest and arid soils. The effects of contaminants are topic of different studies: in some of them contaminants were present in a natural way (e.g., heavy metals in serpentine soils), in others they have been human-introduced (e.g., petroleum).

The ability to compare studies could shed light on many soil processes, identify new insight, discover similar communities around the world, explore and understand the soil biodiversity, but this is at the moment very complicated due to lack of standards in the field both for the soil and the proteomics data. Even though many studies have reported the soil properties, the way to report them has not been consistent across the papers and there is no standard framework that researchers can use to compare similarities and differences between the studied soils. Comparative studies enable a deeper understanding of the soil physical and chemical properties. One interesting resource for comparison of studies is the Paleontology, Geobiology and Earth Archives Research Center (PANGEA) (http://www.pangea.unsw.edu.au/research) that has already emphasized the process of discovery and integration of ideas in different areas such as landscape evolution. This resource has allowed to create a standard framework, to understand the range of natural variability present in biological systems, enhancing the capacity to discriminate natural cycles from recent human perturbations.

Concerning the proteomics data, currently not many scientists release upon publication the raw data underlying their experiment and on which they have built their conclusions. On the other hand, many publishers require as mandatory the raw data deposit in the guidelines, but not always this requirement is fulfilled by the authors and checked by the editors. The main repository for proteomics data, since 2012, is the proteomeXchange (PX) consortium (Vizcaíno et al., 2014). The aims of the consortium are the data submission standardization and the dissemination of the proteomics data. Today the consortium includes different repositories from different countries and institutions, with different proteomic targets. The members of the consortium are: PRIDE, PASSEL, MassIVE, jPOST, iProX, Panorama Public, and Peptide Atlas and their main targets are: universal archive, Re-analysis, focused archive, Universal archive, Re-analysis, Universal archive, Universal archive, and focused archive (Deutsch et al., 2020), respectively. The submission process requires several details, including data and metadata. First of all, the authors have to provide the raw data (mandatory) and the derived peak list (optional). Secondly, experimental and technical metadata have to be provided; they are slightly different among the diverse members of the consortium but with a sufficient information to fulfill the requirements for the PX XML format file (http://www.proteomexchange.org/docs/guidelines_px.pdf). Finally, the processed results should be provided including the peptide and the protein identifications (mandatory) and quantification results (optional at present). Currently, two types of submission are supported: complete or partial. The former allows to connect, through PX resource, the identification data to the corresponding mass spectra. The latter provides all the submitted files for download, but it is not possible to parse, integrate and visualize the identification and/or connect the processed results to the corresponding mass spectra (Deutsch et al., 2020).

Concerning the proteomics data released, there are only 17 datasets related to “metaproteomics and soil” in PRIDE (https://www.ebi.ac.uk/pride/archive/) (Martens et al., 2005), among them 11 are associated to a paper, while seven are deposited without any link to a published paper, meaning that only a very small proportion of the authors deposited the raw data in public repositories. The sampling sites related to the data deposited on PRIDE span from Spain to Antarctica and from Northern California to Sweden, indicating the absence of geographical bias and a great variability as spatial coordinates. The act of publishing without depositing the raw data limits other researchers from performing comparative studies. We strongly believe that this restricts the progress, discovering better insights and potential application of metaproteomics field. Publishing the raw data should be made mandatory, both for open and reproducible science, and for allowing the data reuse and exploring new insights. The lack of raw data on repositories is a problem that also concerns other fields, such as studies carried out on gut, water and so on, as shown by the outputs provided by a search of these studies on Web of Science, Pubmed, and Scopus databases and of their deposited raw data on PRIDE (Table 1).

**Table 1:** Papers and raw data published in 2019: paper number results from Web of Science, PubMed and Scopus searches using as topics the two first columns.

**Term 1**	**Term 2**	**Web of science**	**Pubmed**	**Scopus**	**Raw data (PRIDE)**
Metaproteomics	Soil	15	10	13	3
Metaproteomics	Compost	1	0	0	0
Metaproteomics	Sediments	3	3	4	3
Metaproteomics	Gut	32	28	28	7
Metaproteomics	Water	7	4	10	6
Metaproteomics	Air	2	2	2	1
Metaproteomics	Feces	2	3	12	0
Metaproteomics	Lakes	0	1	1	1

*Raw data numbers result from PRIDE query using the terms in the two first columns. All searches were limited to paper from 2019. The term “sediments” include both terrestrial and aquatic ones*.

The aim of this opinion is not only to report the low percentage of dataset related to the soil metaproteomic studies, but also to point out the still concealed potential of the technique if flanked by a proper repository of data. The possibility of comparing studies could shed light on many soil processes, but this is very complicated due to a lack of shared proteomics data. Only 6 (5%; 3 out of the 9 papers cited on PRIDE are not found on WOS) out of 148 studies uploaded the raw data to PRIDE; moreover the remaining studies not always included in the supplementary materials the complete list of identified proteins. On the other hand, many studies provided detailed information about the soil composition, but unfortunately, not in a standardized way. The lack of shared proteomics data, and at the same time the lack of standard metadata on the soil composition, render the different comparative studies a complicated challenge. Owing on the previous considerations, we strongly advise the metaproteomics community to adopt standardized soil metadata, to publish the raw data on PRIDE and to follow the procedures pointed out by ProteomeXchange Consortium. Standardized soil metadata could follow the checklist (Table 2) we have extrapolated by the useful guide for soil describing and sampling, proposed by Schoeneberger et al. (2012).

**Table 2:** Checklist of standardized soil metadata according to Schoeneberger et al. (2012).

**Sample**		**Sampling time**	**Location**			**Element content**		
Name	Replicate	Date	GPS coord	Altitude	Place name	C%	N%	pH
**Soil**			**Land coverage**			**Vegetation**		
Clay %—sand %—silt %	Soil classification	Contaminants	Moisture	Temperature regime	Type of land coverage	Plant common names	Plant scientific names	Vegetation coverage %
**Collection methods**			**Storage system**			**Note**		
Sampling method	Depth of sampling	Unit	Soil treatment (sieved…)	Storage temperature	Container type	Additional information		

These two simple good practices will massively increase the ability to compare studies and carry out bioinformatic analyses, using already published data. Although this opinion focuses on soil metaproteomics data, we hope it will ring a bell for scientists involved in other disciplines and ecosystems.

## Author Contributions

MC, EZ, and AM conceived the idea. MC and EZ designed the structure of the manuscript and drafted it. AM has critically read, corrected, and covered the costs to publish in open access.

### Conflict of Interest

The authors declare that the research was conducted in the absence of any commercial or financial relationships that could be construed as a potential conflict of interest.
